# Supplementary material for: A two-step deep learning method for 3DCT-2DUS kidney registration during breathing
Source: Sci Rep. 2023 Aug 8;13:12846. doi: 10.1038/s41598-023-40133-5 (PMC10409729; doi:10.1038/s41598-023-40133-5)
Supplement: Supplementary file 1 — Supplementary Information. [file 41598_2023_40133_MOESM1_ESM.pdf]

Appendix

A: Feature Network

The ULBNet is a 5-level U-Net with residual block replacing the original convolutional layer (Fig.1).

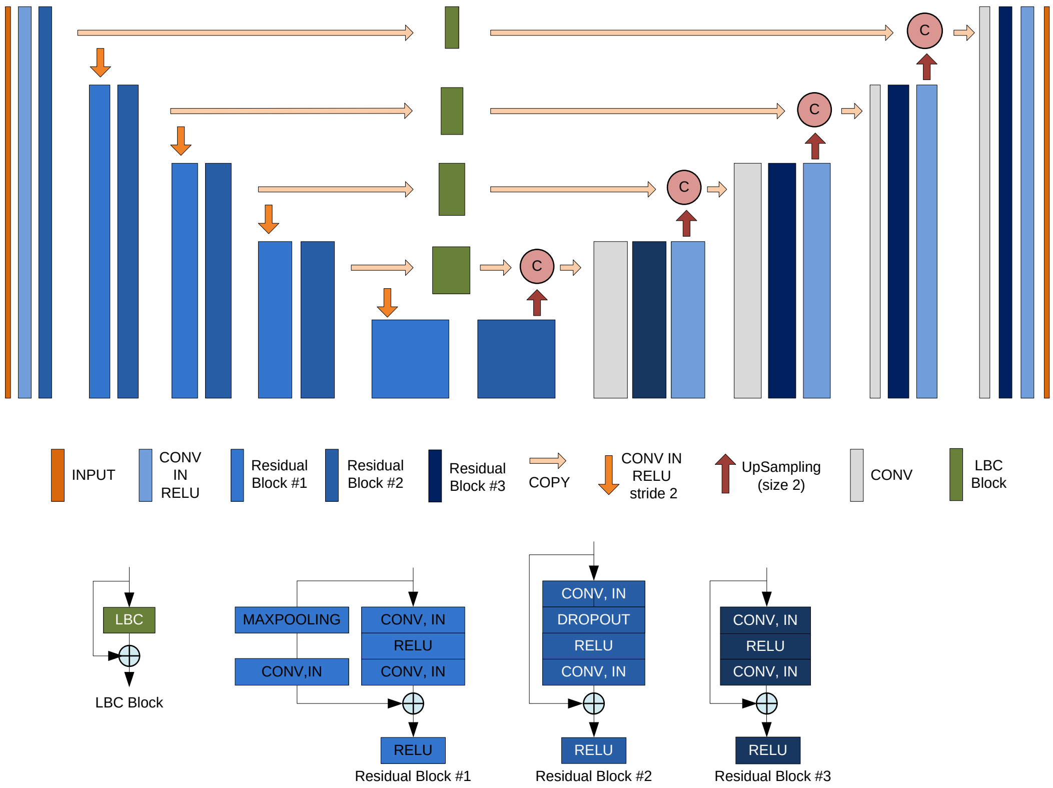

Figure 1. The 3D ULBNet network architecture.

B: Reference Plane

For each U/S frame, a manually selected reference CT cutting plane with the overlap of kidney boundaries was displayed side-by-side to four experienced clinicians to unanimously verify if they

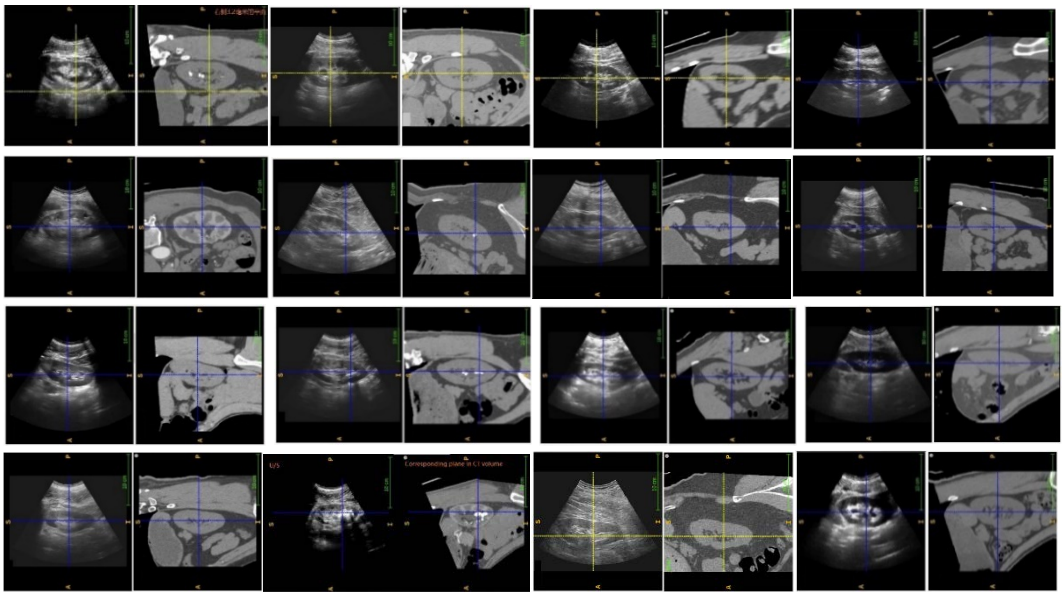

Figure 2. Examples of the verified reference planes.

were the same cutting plane of the kidney. The verified planes constructed our basic reference set, from which we extended the training set. There were 22 out of 25 pairs of CT volume and U/S sequences verified by clinicians that their 3D cutting plane in CTs was the same as that in U/S images (Fig. 2).

C: Transformation Parameter Estimation

Six parameters in transformations that resulted in the reference CT planes from initial positions (Fig. 3) were modelled in 2-sigma Gaussian distributions.

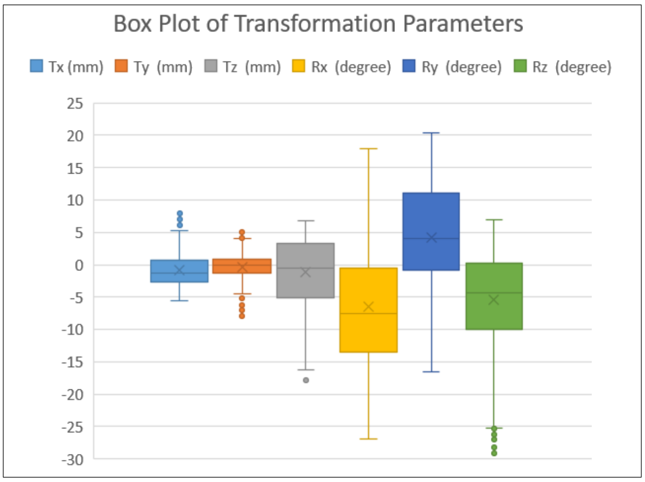

Figure 3. Distribution of transformation parameters (T: translation, R: Euler angle)

D: Generated Datasets

The generated datasets were only used for training. The more datasets generated, the smaller the MCD achieved (Fig.4). A local minimum occurred at 6000 pairs. The global minimum occurred approximately 12,000 pairs, where the 3D CT-CT distance was close to 2D CT-US distance. Thus, we generated approximately 12,000 training data pairs, 10 times of clinic datasets, to pretrain the registration network.

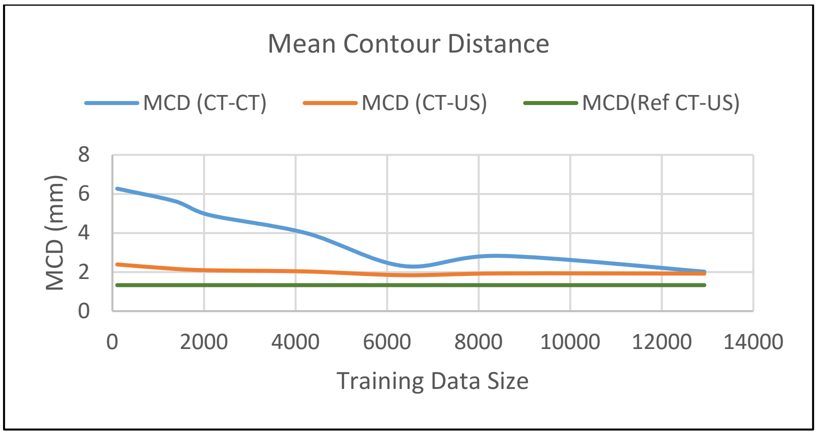

Figure 4. The average MCD decreases when the generated training datasets increase.

E: U/S Feature Network Performance on Window Size

We tested feature extraction on U/S image sequences with different window sizes (Table 1). The best performance was obtained at a size of 5.

Window size	DICE	Sensitivity	Specificity
1	0.9391	0.9492	0.9455
3	0.9543	0.9714	0.9407
5	**0.9639**	**0.9736**	0.9560
7	0.9604	0.9675	**0.9561**
9	0.9590	0.9656	0.9544

Table 1: Feature network performance on kidney segmentation in U/S images with different window sizes. The U/S images were of dimension 256×192×WindowSize.

F: Registration Performance on Transfer Learning Strategy

The model performance on four test sequences from the second cycle was evaluated. Overall, one-cycle training performed best, as observed in Table 2. Moreover, we found that transfer learning helped to achieve a lower loss than the model trained from scratch, as shown in Table3.

	Metric (mm)		Learning strategy			
			Pretrained model	One-shot (5epoches)	Five-shot (5epoches)	One-cycle (2epoches)
Case 01	CT-US	HD	7.25±1.22	3.99±0.86	3.20±0.72	3.01±0.73
		MCD	2.53±0.30	1.07±0.32	0.85±0.18	0.75±0.17
	CT-CT	HD	5.78±1.50	6.12±1.66	6.00±1.70	6.75±2.42
		MCD	2.03±0.56	2.06±0.56	1.80±0.52	2.17±0.95
Case 02	CT-US	HD	8.32±1.21	4.91±0.77	4.35±1.22	4.16±0.65
		MCD	2.11±0.46	1.61±0.70	0.95±0.20	0.85±0.13
	CT-CT	HD	19.18±2.66	4.52±1.34	4.80±1.69	4.45±2.09
		MCD	6.38±0.84	2.00±1.01	1.56±0.62	1.47±0.91
Case 03	CT-US	HD	5.39±0.72	4.89±0.90	3.61±0.99	3.54±1.11
		MCD	1.50±0.19	1.36±0.38	0.95±0.27	0.79±0.19
	CT-CT	HD	16.52±1.21	11.92±2.32	4.68±1.44	2.72±0.64
		MCD	5.70±0.39	3.93±0.82	1.64±0.84	0.58±0.26
Case 04	CT-US	HD	5.89±1.11	5.29±1.16	4.45±1.10	4.52±0.99
		MCD	1.99±0.23	1.75±0.28	1.43±0.23	1.40±0.23
	CT-CT	HD	4.90±0.92	4.68±0.85	2.94±0.79	1.98±0.34
		MCD	2.27±0.84	2.00±0.86	0.89±0.45	0.39±0.19
Average	CT-US	HD	6.71±1.06	4.77±0.92	3.90±1.00	3.80±0.87
		MCD	2.03±0.29	1.44±0.42	1.04±0.22	**0.94±0.18**
	CT-CT	HD	11.59±1.57	6.81±1.54	4.63±1.40	3.97±1.37
		MCD	4.09±0.65	2.49±0.81	1.47±0.60	**1.15±0.57**

The CT-US distance is calculated between kidneys boundaries on CT and U/S. The CT-CT distance between kidney contours on resulted CT plane and reference CT plane.

Table 2: 3DCT-2DUS kidney registration performance on four learning strategies.

	Loss (Epoch = 2)		$Loss_{min}$	
	TFL	TFS	TFL	TFS
Case 01	-0.9744	-0.9681	-0.9773	-0.9762
Case 02	-0.9642	-0.9508	-0.9689	-0.9673
Case 03	-0.9624	-0.9507	-0.9706	-0.9699
Case 04	-0.9610	-0.9559	-0.9692	-0.9683
Average	**-0.9655**	-0.9563	**-0.9715**	-0.9704

TFL: training model via transfer learning, TFS: training model from scratch, $Loss_{min}$: the minimal loss obtained within 100 epochs.

Table 3: Feature-image-motion loss obtained from two learning strategies: TFL and TFS.

G: Registration Network Performance on Window Size

We tested registration on motion regularisation with window sizes of 1, 3, and 5 (Table 4), and a size of 1 resulted in best accuracy, while a size of 5 resulted in visually smoother CT cutting plane sequences. It was reasonable that the large window size gave a smooth regularisation at the expense of accuracy. The size of 1 approached the global minimum by free transition and rotation, while the size of 5 approached the optimal transformation by transition on a larger scale due to motion regularisation. Since smoothness was also important, we selected 5 to achieve overall optimality.

WindowSize	Metric (mm)	CT-CT	CT-US
1	HD	2.91±1.11	3.73±0.95
	MCD	**0.97±0.44**	0.95±0.21
3	HD	3.39±1.38	3.82±0.96
	MCD	0.99±0.58	0.98±0.25
5	HD	3.97±1.37	3.80±0.87
	MCD	1.15±0.57	**0.94±0.18**

Table 4: CT-US registration performance on the number of consecutive frames (WindowSize) used for motion regularisation.

H: Uncertainty Estimation

Due to the unavailability of ground truth transformations for image registration, an uncertainty estimate would help gauge system reliability. Registration uncertainty was estimated by randomly removing 1~10% training datasets and estimating the standard deviation of the performance. The experiment was repeated (n=10). The uncertainty in MCD was approximately 1 mm for the CT-CT distance and 0.22 mm for the CT-US distance (Table 5).

	CT-US		CT-CT	
	HD	MCD	HD	MCD
Standard deviation (mm)	1.62	0.22	2.97	1.01

Table 5: Performance variance in ten repeated experiments on CT-US registration with random training datasets removed.

I: Registration Results Examples

We illustrated the registration results from the two-step training strategy by plotting the U/S image plane in CT volume (Fig. 5). The initial position (blue), the reference plane position (green), and the result plane position (red) can be observed in the coronal view. Our method resulted in the closest cutting plane to the reference plane compared to the other methods.

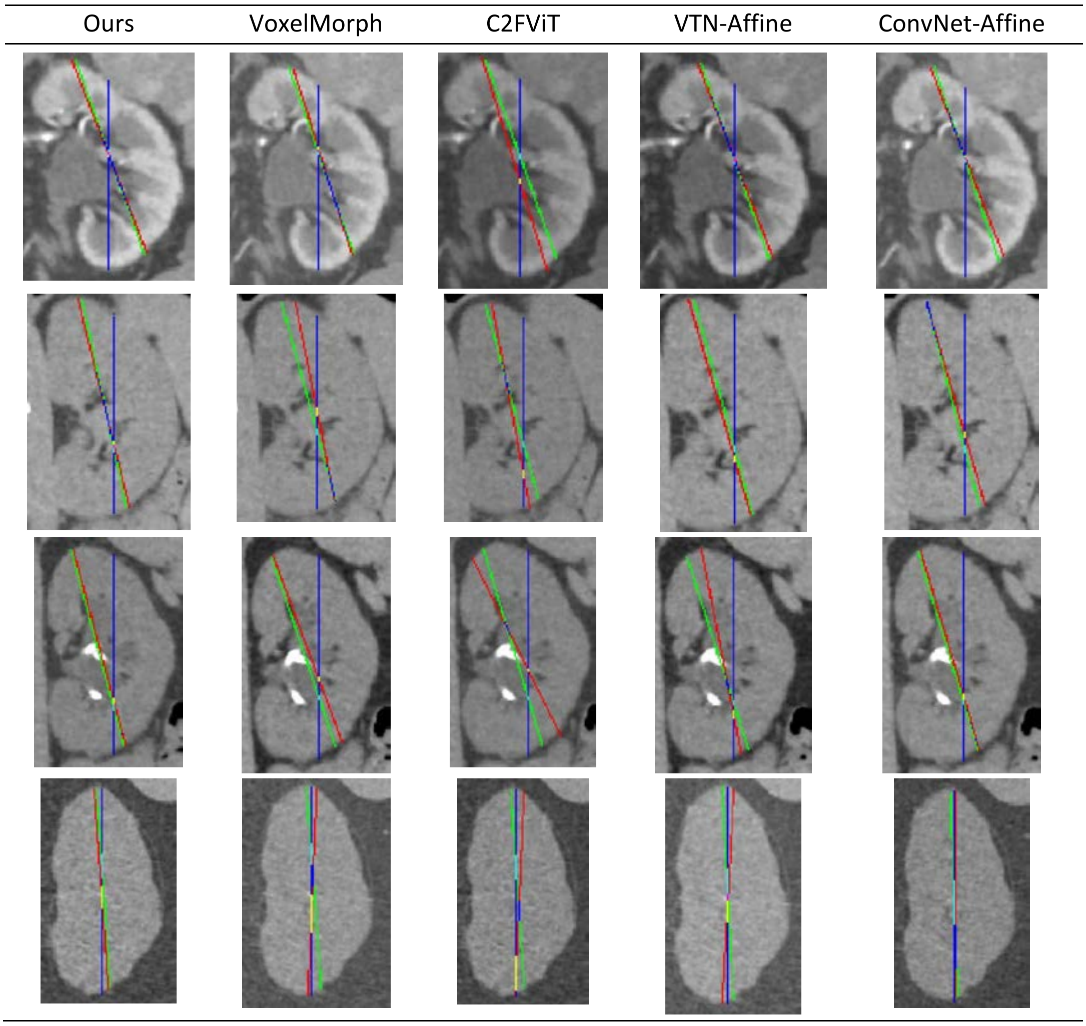

Figure 5. Example of the U/S plane in CT volume, displayed in coronal view. Blue: initial position, green, reference position, red: registration position.
